# Using Bayesian statistics to estimate the likelihood a new trial will demonstrate the efficacy of a new treatment

**DOI:** 10.1186/s12874-017-0401-x

**Published:** 2017-08-22

**Authors:** David J. Biau, Samuel Boulezaz, Laurent Casabianca, Moussa Hamadouche, Philippe Anract, Sylvie Chevret

**Affiliations:** 1 INSERM U1153, Paris, France; 2 Service de chirurgie orthopédique,Hôpital Cochin, 27 rue du faubourg Saint-Jacques, 75014 Paris, France; 3 Université Paris-Descartes, Paris 5, Paris, France; 4 Université Paris-Diderot, Paris 7, Paris, France

**Keywords:** Meta-analysis, Bayesian statistics, Cumulative, Direct probability, Posterior probability, Predictive probability, Superiority

## Abstract

**Background:**

The common frequentist approach is limited in providing investigators with appropriate measures for conducting a new trial. To answer such important questions and one has to look at Bayesian statistics.

**Methods:**

As a worked example, we conducted a Bayesian cumulative meta-analysis to summarize the benefit of patient-specific instrumentation on the alignment of total knee replacement from previously published evidence. Data were sourced from Medline, Embase, and Cochrane databases. All randomised controlled comparisons of the effect of patient-specific instrumentation on the coronal alignment of total knee replacement were included. The main outcome was the risk difference measured by the proportion of failures in the control group minus the proportion of failures in the experimental group. Through Bayesian statistics, we estimated cumulatively over publication time of the trial results: the posterior probabilities that the risk difference was more than 5 and 10%; the posterior probabilities that given the results of all previous published trials an additional fictive trial would achieve a risk difference of at least 5%; and the predictive probabilities that observed failure rate differ from 5% across arms.

**Results:**

Thirteen trials were identified including 1092 patients, 554 in the experimental group and 538 in the control group. The cumulative mean risk difference was 0.5% (95% CrI: −5.7%; +4.5%). The posterior probabilities that the risk difference be superior to 5 and 10% was less than 5% after trial #4 and trial #2 respectively. The predictive probability that the difference in failure rates was at least 5% dropped from 45% after the first trial down to 11% after the 13th. Last, only unrealistic trial design parameters could change the overall evidence accumulated to date.

**Conclusions:**

Bayesian probabilities are readily understandable when discussing the relevance of performing a new trial. It provides investigators the current probability that an experimental treatment be superior to a reference treatment. In case a trial is designed, it also provides the predictive probability that this new trial will reach the targeted risk difference in failure rates.

**Trial registration:**

CRD42015024176.

**Electronic supplementary material:**

The online version of this article (doi:10.1186/s12874-017-0401-x) contains supplementary material, which is available to authorized users.

## Background

For the past decade efforts have been made, both by scientists, journal editors and funding sources, to increase value and reduce waste of medical research [1]. As early as 1996, the CONSORT statement required that data from a new trial should be interpreted “in the light of the totality of the available evidence” [2]. Editors later joined in by asking those submitting RCTs to set the new results in the context of systematic reviews or meta-analyses of the results of any other relevant RCTs [3, 4]. The effect of such a policy has yet to be seen however [5, 6]. Funders are also participating in order to avoid wasting of financial resources. Recently the National Institute for Health Research has stated that it “will only fund primary research where the proposed research is informed by a review of the existing evidence” [7].

Overall, when planning a trial researchers would like to answer three questions given the available previous evidence: the first is “what is the likelihood that the experimental treatment is superior to the control treatment given the evidence accumulated so far?”; the second is “what is the likelihood that a new trial, given some design parameters and previous evidence, will demonstrate the superiority of the experimental treatment?”; and the last is “what are is likelihood that this new planned trial shifts the overall evidence accumulated in the literature?”. To summarize evidence from the literature, meta-analyses are used, based on fixed or random-effect models. In the fixed-effects model, the parameter of interest is assumed to be identical across studies and the difference between the observed proportion and the mean is only due to sampling error. In the random-effects model, the observed difference between the proportions and the mean cannot be entirely attributed to sampling error but may rely to other unobserved factors. However, these common frequentist’s approaches are unable to answer such important questions. By contrast, Bayesian statistics, that also consider the parameter of interest as a random variable, and combining accumulated evidence from different sources, appear to fit naturally this situation. Indeed, it provides direct probability statements with regards to effect sizes and/or predictive distributions. These probabilities, for instance the likelihood that an experimental treatment is superior to a control treatment, can then be used to appreciate how any future trial would change the conclusion of the available literature [8]. In the specific setting of cumulative meta-analyses with recursive access to information, Bayesian approaches appear quite appropriate to identify the time when another trial becomes superfluous.

For instance, a current question among the orthopaedic community is whether patient-specific instrumentation, a recent innovative technology used during total knee replacement to improve implant positioning, is superior to conventional instrumentation [9]. To ensure the long term success of a knee replacement it is paramount that the best limb alignment (180°) is achieved during the operation: knees which deviate by more than 3° from this angle are more likely to fail early. The standard procedure to ensure limb alignment is to use intra-medullary jigs. Recently, patient specific guides based on a preoperative scanner or MRI have been developed to improve the precision of limb alignment during the surgery. Seventeen studies and 10 reviews or meta-analyses have been published to address the issue in less than 4 years and there is still no evidence for a difference between both treatments.

We therefore conducted a Bayesian cumulative meta-analysis of patient-specific instrumentation compared to conventional instrumentation in patients undergoing total knee replacement. We first estimated the probability that the experimental treatment is superior to the control treatment cumulatively through time given the evidence accumulated. We then estimated the probability that a new fictive trial, based on previous evidence, would demonstrate the superiority of the experimental treatment. Last, we estimated the design requirements for a trial to change the overall evidence accumulated.

## Methods

### Protocol and registration

Eligibility criteria, information sources, data items and methods of the analysis were specified in advance and documented in a protocol. The protocol was registered at PROSPERO (protocol registration number: CRD42015024176). PRISMA guidelines were followed [10].

### Eligibility criteria, information sources, and search strategy

Two reviewers (SB, LC) independently evaluated studies for eligibility; disagreements between the reviewers were resolved by consensus, and if necessary, by consultation with a third reviewer (DB). Randomized clinical trials studying conventional versus patient-specific instrumentation for total knee replacement were eligible. We considered studies including participants of any age, undergoing total knee replacement for any reason. Conventional instrumentation included intra- or extra-medullary alignment techniques; patient-specific instrumentation included CT- or MRI-based preoperative planning. Total knee replacements performed with computer navigation were excluded. The primary outcome measure was the proportion of failures. Failures were defined as patient with a frontal alignment departing from neutral by more than three degrees, in varus or valgus. No restriction was made on the method used for measuring the frontal alignment.

Publication in English, until January 1st, 2016 were examined. Studies were identified by searching Medline via PubMed, EMBASE and the Cochrane library. The last search was performed on May 1, 2016. Two authors (SB, LC) selected eligible studies first on titles and abstracts and then on full text for selection criteria. Finally, the references of included studies were hand searched in order to detect additional studies. We excluded duplicate reports, pilot studies, and abstracts from meeting proceedings unless published as full-text reports in a peer-reviewed journal, given concerns related to their small sample size and study design. We used, in various relevant combinations, keywords pertinent to the groups and intervention of interest: custom-fit, custom, patient-specific, psi, patient specific instrumentation, and knee replacement.

### Data items and risk of bias in individual studies

Two investigators (SB, LC) independently extracted data from the primary texts, Appendix and Additional file 1, using a data abstraction sheet that contained fields for: first author name, trial name, year of publication and recruitment period, number of patients in each treatment group, crossover, imaging method used for preoperative planning in the patient-specific instrumentation group and surgical technique used in the conventional instrumentation group, number of failures, details regarding trial design. Disagreements were resolved by consensus, and if necessary, after consultation with a third and fourth reviewer (DB, MH). The risk of bias in individual studies was assessed at the outcome level using the Cochrane collaboration’s tool [11]. Authors were contacted to provide additional information when relevant.

### Summary measures, synthesis of results, and risk of bias across studies

Bayesian meta-analysis was performed cumulatively, on trials ordered according to publication time (Entrez date on PubMed). The risk of publication bias was assessed by funnel plots of effect estimates against sample size [12]. Consider K comparative studies reporting summary binary outcomes. The data from each study, j = 1, …, K, constitutes a pair of independent binomial variables, X_1J_ and X_2j_, the number of events out of n_1_ and n_2_ subjects in the treatment and control arms.: X_1j_ ~ Binom (n_1_, p_1j_) and X_2_j ~ Binom (n_2_, p_2j_), where p_ij_ for *i* = 1, 2 are the risks in the treatment and the control arm, respectively. In a Bayesian framework, the proportion of failures p_ij_ was modelled through a beta-binomial model in each randomized arm (*i* = 1, 2), separately. Our prior information with regards to each of these proportions is formalized by a prior that is then actualized along the meta-analyses into a posterior distribution. Indeed, the beta distribution is the conjugate prior distribution for the parameter if the data are binomial, so that the posterior is still a beta distribution. First, non-informative Beta priors (i.e., uniform priors), were used to represent the large uncertainty with regards to the outcomes before any published trial data. Then, the posterior distributions computed after the trial, were used as the priors for the next trial, and so on. We defined the posterior probability that the failure proportion in the experimental arm is below that in the controls, namely the risk difference, as the treatment effect measure. The risk of bias across studies was assessed visually for each of the seven Cochrane collaboration’s items [11].

First, we estimated the posterior probabilities that the proportion of failures in the experimental group was below that observed in the control group by 5 and 10% according to the accumulated evidence, i.e. after the inclusion of each new trial in the cumulative meta-analysis; such values were considered of clinical importance in this particular setting. We then computed the predictive probabilities that given the results of all previous published trials (a priori information), the next scheduled trial would achieve a risk difference of observed failure rates of at least 5% or 10% in favour of the experimental treatment. We also assessed the likelihood that a new planned trial shifts the overall evidence accumulated in the literature, by simulating samples of patients with response rate in the control arm drawn from the last posterior (obtained at the end of the meta-analysis), with varying sample sizes and failure probability in the intervention arm, then computing the posterior probability of reaching a difference of at least 5% between arms.

We finally computed the required sample size of a new trial to reach a 95% coverage probability on average for the posterior credible interval (CrI) of 5% length for the risk difference. All point estimates are presented with 95% CrI and were computed using Markov Chain Monte Carlo (MCMC) simulation (see Additional file 1 for details).

As a sensitivity analysis, frequentist cumulative and non-cumulative standard random effects meta-analyses were also performed. We used a Binomial-normal model that imposed a normal distribution on log-odds odds in treatment and control arms to incorporate the between-studies heterogeneity. Estimates of the risk difference were obtained from random-effect models using the DerSimonian and Laird method [13], with 95% confidence intervals. According to the Cochrane principles, a value of 0.5 was added to arms where no failure occurred; trials where no failures occurred were excluded from the analysis. I^2^ was used to quantify heterogeneity and we used the Q chi-squared statistic to test heterogeneity across trials with *P* < 0.1 being considered significant.

All computations were performed on R version 3.2.2 (https://www.R-project.org/), using the R2jags (https://cran.r-project.org/web/packages/R2jags/) and rmeta (https://cran.r-project.org/web/packages/rmeta/) packages.

### Patient involvement

No patients were involved in setting the research question or the outcome measures, nor were they involved in developing plans for design or implementation of the study. No patients were asked to advice on interpretation or writing up of results. There are no plans to disseminate the results of the research to study participants or the relevant patient community.

## Results

### Study selection, study characteristics, results of individual studies, and risk of bias within studies

Thirteen trials, published between 2013 and 2015, were identified and used, based on complete text review (Appendix Figure 4) [14–26]. Overall, 1092 patients with a mean age of 68 [67–70] years old and a mean BMI of 29.5 [28.5–30.5] were included, 554 in the experimental group and 538 in the control group (Table 1). Six studies used MRI for preoperative planning, five CT, and two studies used both (when necessary, groups were pooled to avoid the duplicate counts of patients). Individual risk differences ranged from −25 to +18.5% (Appendix Figure 5a). Due to the procedure evaluated, the risk of bias was significant for all individual studies with regards to blinding since surgeons could not be blinded; however, for most studies, the outcome assessor was blinded (Table 1). Based on the funnel plot of the effect sizes of all studies we did could not identify any serious evidence in favour of a publication bias (Appendix Figure 6).

**Table 1:** Description of the retrieved trials

First author	e-date	Imaging	Female/male^a^	Age (mean)	BMI (mean)	RSG	AC	BOPH	BOA	IOD	SR	OB
Chareancholvanich	02/03/2013	mri	70/10	70	28	low	uk	high	low	low	low	uk
Victor	26/04/2013	mri/ct	86/42	67	-	uk	low	high	low	high	low	high
Roh	03/08/2013	ct	82/8	70	27	low	low	high	uk	high	low	uk
Hamilton	06/08/2013	ct	31/21	68	31	uk	uk	high	low	high	low	uk
Boonen	10/08/2013	mri	106/74	67	30	low	low	high	low	low	low	low
Parratte	15/08/2013	mri	24/16	71	29	high	low	high	low	low	low	uk
Chotanaphuti	04/09/2013	ct	70/10	70	25	uk	uk	high	low	low	low	low
Woolson	07/03/2014	ct	0/63	66	33	low	low	high	low	high	low	low
Kotela	28/06/2014	ct	66/29	67	30	uk	uk	high	low	low	low	low
Pfitzner	16/07/2014	mri/ct	51/39	65^b^	30	low	low	high	low	low	low	low
Yan	14/09/2014	mri	41/19	69	-	low	uk	high	low	low	low	uk
Abane	09/01/2015	mri	88/52	69	29	low	uk	high	low	high	low	low
Molicnik	04/03/2015	mri	31/7	67	33	uk	uk	high	uk	uk	low	uk

*RSG* random sequence generation, *AC* allocation concealment, *BOPH* blinding of participants/care providers, *BOA* blinding of outcome assessors, *IOD* incomplete outcome data, *SR* selective reporting, *OB* other biases. ^a^ sex ratio in shown for patients randomized and outcome is shown for patients analyzed, therefore numbers may differ. ^b^ mean of group’s median

### Synthesis of results, and risk of bias across studies

**Fig. 1:**
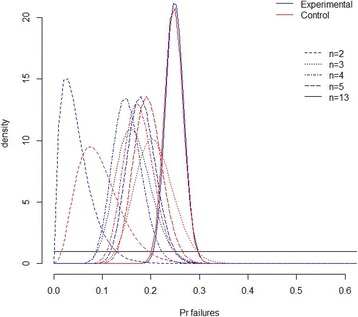
Evolution over the meta-analysis of the uncertainty in the proportion of failures in both arms, as quantified by the prior (*black line*) then actualized into a posterior distribution after *n* = 2, 3, 4, 5, and 13 trials

**Fig. 2:**
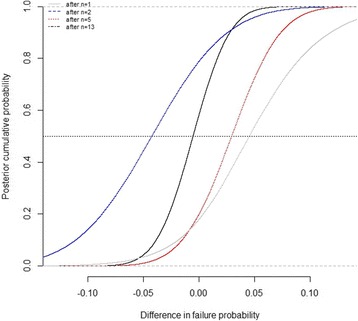
Posterior distribution function of the difference in failures rates across arms; for instance, there was a 0.042 posterior probability, after the results of the 13 trials that the failures rate in the experimental was below that of the control by 5%

Based on the information accumulated after the last trial, the mean posterior estimates of failure probabilities in each group evolved over time to stop at 24.1% (95% CrI: 20.7%; 27.7%) in the experimental arm compared to 24.6% (95%CrI: 21.0%; 28.4%) in the control arm (Fig. 1; Table 2). In other words, patient-specific instrumentation decreased the estimated proportion of failures by 0.5% (95% CrI: −5.7%; +4.5%) (Fig. 2; Table 2); there was no important difference with the pooled estimate obtained from the sensitivity frequentist random-effect meta-analysis (Appendix Figure 5b). The risk of bias across studies was, by design, maximum for blinding of care providers, possibly significant for the randomisation and allocation concealment procedures (Appendix Figure 7).

**Table 2:** Estimates of the risk difference

FirsFirst author	e date	Nb	Events	Nb	Events	Cumulative Evidence						Fictive Trial		
						Mean posterior estimates of outlier probabilities		Estimated risk difference^a^	(95% CrI)	Pr. risk diff. Above		Additional planned sample size	Predictive probability that proportion of failures in the next sample was in the treated compared to the control	
		Control		Treated		Control	Treated			5%	10%		5% less	10% less
Chareancholvanich	2013–03-02	40	3	40	1	0.0952	0.0476	−0.048	(−0.161; +0,059)	0.464	0.16	-	48.5%	25.6%
Victor	2013–04-26	64	18	61	15	0.2075	0.1650	−0.042	(−0.100; +0.242)	0.444	0.14	125	47.5%	24.0%
Roh	2013–08-03	48	5	42	5	0.1753	0.1517	−0.024	(−0.107; 0.059)	0.267	0.036	90	33.3%	9.8%
Hamilton	2013–08-06	26	8	26	9	0.1944	0.1813	−0.013	(−0.095; 0.069)	0.187	0.018	52	26.3%	6.6%
Boonen	2013–08-10	82	15	86	26	0.1908	0.2218	0.031	(−0.038; 0.101)	0.011	0.00012	168	5.4%	0.5%
Parratte	2013–08-15	20	2	20	4	0.1844	0.2202	0.036	(−0.031; 0.102)	0.0057	0.00003	40	3.8%	0.2%
Chotanaphuti	2013–09-04	40	5	40	2	0.1770	0.1987	0.022	(−0.039; 0.083)	0.010	0.00004	80	5.2%	0.3%
Woolson	2014–03-07	26	10	22	9	0.1925	0.2124	0.020	(−0.039; 0.081)	0.011	0.00005	48	5.6%	0.3%
Kotela	2014–06-28	46	14	49	24	0.2056	0.2474	0.042	(−0.016; 0.101)	0.001	0.000001	95	1.5%	<0.0%
Pfitzner	2014–07-16	30	13	60	11	0.2217	0.2388	0.017	(−0.039; 0.073)	0.009	0.00002	90	4.9%	0.2%
Yan	2014–09-14	30	13	30	8	0.2357	0.2406	0.005	(−0.050; 0.060)	0.024	0.00009	60	8.4%	0.4%
Abane	2015–01-09	67	22	59	19	0.2476	0.2495	0.002	(−0.050; 0.054)	0.025	0.00006	126	8.6%	0.4%
Molicnik	2015–03-04	19	4	19	0	0.2463	0.2410	−0.005	(−0.057; 0.045)	0.042	0.00013	38	11.2%	0.5%

*Negative values favour the experimental treatment. Number of patients in the control (n. ctr) and experimental (n. exp) groups; number of events (ev. ctr and ev. exp); credibility interval (ctrCrI); probability (Pr)

Estimation of the probabilities that the proportion of outliers in the experimental group is below that observed in the control group by 5 and 10% according to the accumulated evidence. Estimation of the Bayes predictive probability that the risk difference be of at least 5 and 10% in favour on the experimental group

### What is the likelihood that the experimental treatment is superior to the control treatment given the evidence accumulated so far?

The posterior probabilities that the proportion of failures in the experimental group be inferior to the proportion of failures in the control group by 5 and 10% was less than 5% after trial #4 and trial #2 respectively. After all the available evidence, these probabilities were 4.2 and 0.013% (Table 2). The likelihood that the experimental treatment is superior to the control treatment is therefore marginal at best.

### What is the likelihood that the next trial, given some design parameters and previous evidence, will demonstrate the superiority of the experimental treatment?

When designing a new trial according to the evidence accumulated previously, we computed that the predictive probability that the failure rate was below that observed in controls by 5% dropped from 45% after the first trial down to 11% after the 13th; when considering difference of at least 10%, these figures decreased from 21 to 0.5%, respectively (Table 2). This argues in some sense that the likelihood of any consequent benefit for patients in the new trial is rather low.

### What is the likelihood that a new planned trial shifts the overall evidence accumulated in the literature?

**Fig. 3:**
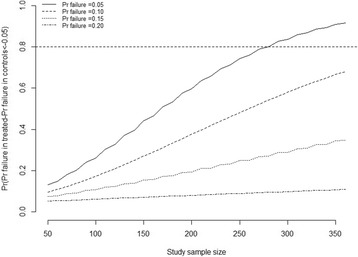
Predictive probabilities that a new 14th fictive simulated trial shifts the evidence in favour of the experimental group according to various design parameters

Given the evidence provided by the meta-analysis, that is a 24.1% of failures in the experimental vs. 24.6% in the control arm, the predictive probabilities that a new 14th fictive simulated trial shifts the evidence in favour of the patient-specific instrumentation were rather small unless the number of patients included were large and the proportion of failures in the experimental group low compared to previous estimates (Fig. 3).

## Discussion

The increasing number of randomized controlled trials (RCT) reported in the medical literature is associated with redundancy [27]. Scientists, journal editors and funding sources, are increasingly aware of this issue and actions are gradually being taken in order to reduce the waste of medical research [1, 3, 4, 7]. Using Bayesian inference we were able to answer three important questions an investigator should ask when considering another trial. In the present case we showed that, after the fourth trial, the probabilities that the experimental treatment decreases the proportion of failures by 5% was less than 20%, and by 10% less than 2%. After the next trial, these proportions dropped to 1% or less erasing all hopes of ever demonstrating the superiority of the experimental treatment. Moreover, the planning of another trial at that time, given reasonable design parameters relative to previous trials, had only a 2% probability of demonstrating the superiority of the experimental treatment. Last we showed that only an overly-optimistic effect size can pretend changing the evidence accumulated after the last trial.

Our work has several limitations. First, the analyses presented are performed on the basis that the evidence published is appropriate, based on quality-based trials. We only considered RCTs while pooling results from RCTs and nonrandomized studies using Bayesian methods appears promising [28]. Moreover, initial evidence is sometimes unreliable, for various reasons, and initially favourable or unfavourable results can later be challenged [29]. Investigators could therefore plan a trial contrary to the evidence accumulated. Second, trialists and methodologists usually see the evidential landscape from a different perspective. The former will think their study is unique and target usually large effect sizes contrary to what is suggested by the evidence accumulated by the latter [30]. Numerous reasons, beyond optimism bias, can explain this discrepancy, such as differences in patient selection and improvements of the experimental treatment. Last, we only considered beta-binomial models for each trial arm, assuming independence between the failures from the two arms of each trial, while joint model such as that proposed by Sarmanov could have been used to handle potential sources of correlation within each trial [31]. Note also that beta-binomial model has been recently reported as a feasible alternative to the standard random-effects model for meta-analysis of odds ratios [32].

Frequentist methods have limited reach to help investigators in deciding if the trial they plan is relevant. Practitioners and methodologists will usually get an approximate answer to this question by looking at, or performing, an up-dated meta-analysis of all previous published trials to yield a pooled estimate of the treatment effect. This is, however, rarely done [30, 33, 34]. The first reason for this failure may be that the pooled estimate of treatment effect does not speak to a practitioner enough, all the more when the volume of the evidence accumulated is small. For instance, in the present cumulative meta-analysis, after the fourth trial, the estimation of the risk difference was −0.013 (95% CrI -0.095; 0.069) in favour of the experimental treatment. Given the rather centred pooled estimate and its credible interval, one could see that evidence as inconclusive; effect sizes of 5 and 10% in favour of the experimental treatment could be deemed reasonable [35]. On the contrary the Bayesian approach provides a quite readily understandable quantity: there is little chance that the experimental treatment is superior to the control treatment. The second reason is that trialists looking at the previous evidence in a frequentist perspective to define design parameters are likely to succumb to an optimism bias, namely the unwarranted belief in the efficacy of new therapies, and hence distort the planning of the trial in favour of the experimental treatment [36]. In a retrospective analysis of 359 trials Djulbegovic and colleagues showed that investigators consistently overestimated the expected treatment effect and this was more pronounced for inconclusive trials [35]. On the contrary, the formal use of prior information in a Bayesian framework could help in reducing this optimism bias. In the present study, given the evidence accumulated so far and the sample size used by investigators, the probabilities that the planned trial had more than a 80% chance to demonstrate an effect size of at least 5% quickly fell below 10%.

The Bayesian approach provides readily usable probabilities for clinicians and policymakers. With those, they can directly appreciate the relevance of a new trial, the probability that this trial will succeed, and how likely it is to change the evidence accumulated so far. Moreover meta-analyses are often performed once a relatively important amount of evidence has been reached, while one may wish to repeatedly perform cumulative analysis of all the trial data accumulated starting at an early time. In this framework, Bayesian methods are particularly useful given their natural fit to sequentially accumulated data and their direct translation in terms of probability statements with regards to the effect size. The Bayesian approach should not be seen in opposition to the more common frequentist approach but more so as a complementary viewpoint. If used appropriately, it could help clinicians designing successful trials early and convince policymakers to abandon the funding of unnecessary later trials.

Although the probabilities presented seem more easily understandable, it remains to be demonstrated that clinicians, investigators, and policymakers are more receptive to those. Frequentist methods provide some help in deciding whether another trial is justified, but they lack clarity [37]. Bayesian statistics have attracted a rather unreasonable mistrust over time and may fail to convince trialists used to look at things from a frequentist standpoint [38]. Future research should aim at measuring how the scientific community is responsive to these estimates.

## Conclusions

Bayesian probabilities are readily understandable when discussing the relevance of performing a new trial. It provides investigators the current probability, that is given all previous evidence, that an experimental treatment be superior to a reference treatment. In case a trial is designed, it also provides the predictive probability that this new trial be successful, namely that it will reach the targeted risk difference in failure rates.

## Figures and Tables

**Fig. 4:**
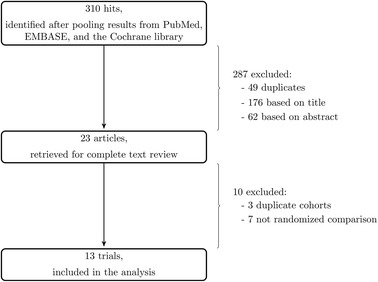
Flow diagram of study selection

**Fig. 5:**
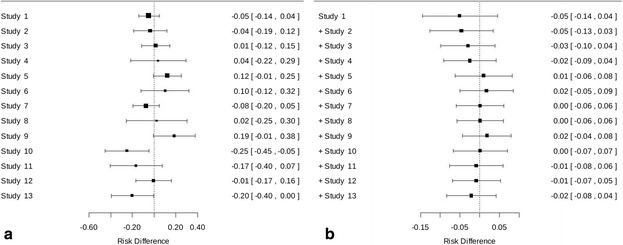
Forest plot of the risk difference in failure rates between the control and experimental groups in a non-cumulative frequentist meta-analysis (I^2^ = 45%, Q = 0.04) (**a**) and in a cumulative frequentist meta-analysis (**b**). Negative estimates are in favour of the experimental group

**Fig. 6:**
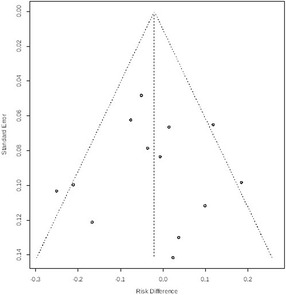
Funnel plots of effect estimates against sample size

**Fig. 7:**
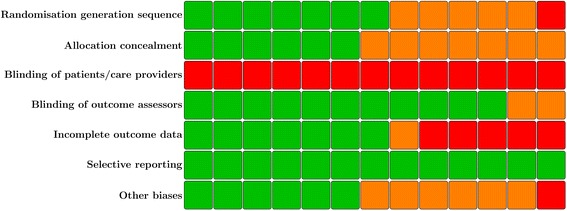
Risk of bias across studies according to the Cochrane collaboration tool [11]

## Additional file

Additional file 1: Bayesian Analyses. (DOCX 21 kb)

## Abbreviations

BMI: Body mass index
CrI: Credibility interval
CT: Computer tomography
IQR: Interquartile range
MRI: Magnetic resonance imaging
RCT: Randomized controlled trial
