# Supplementary material for: Using Bayesian statistics to estimate the likelihood a new trial will demonstrate the efficacy of a new treatment
Source: BMC Med Res Methodol. 2017 Aug 22;17:128. doi: 10.1186/s12874-017-0401-x (PMC5568256; doi:10.1186/s12874-017-0401-x)
Supplement: Additional file 1: — Bayesian Analyses. (DOCX 21 kb) [file 12874_2017_401_MOESM1_ESM.docx]

**Additional file 1- Bayesian Analyses**

Let define the failure as a binary outcome, Y, where Y=1 denotes the failure to treatment and Y=0 denotes the absence of a failure. The observed number of failures among the $n_{k}$ patients allocated to arm *k* is given by$y_{k}=\sum_{i=1}^{n} y_{i}1_{i\in k}$, where $1_{i\in k}$ denotes the indicator function ($1_{i\in k}=1$ if the ith patient has been allocated to arm k, and 0 otherwise).

We used a Bayesian inference framework, where $\pi_{k}=P\left( Y=1 | A=k \right)$ denotes the probability of failure in the arm A=k (k=0, …, K). Using a beta Be($a_{k}$,$b_{k}$) prior for π_k_, the posterior probability of π_k_ is still a beta distribution given by Be($a_{k}$+$y_{k}$,$b_{k}$+$n_{k}-y_{k}$) due to the natural conjugate property of the beta family for binomial sampling. Before the first trial, prior information was considered as negligible so that uniform Beta(1,1) priors were used for $\pi_{k}.$ Then, posterior density after trial J was used as the prior for posterior computations for trial J+1.

The main aim of the cumulative meta-analysis is to, over the publication of K successive trials, quantify the likelihood of the experimental treatment benefit over the control regarding the probability of failure. Let $y_{ki}$denote the number of responses observed in the trial *i* among the $n_{ki}$ patients randomly allocated to arm *k* (k=0,1, where k=0 denotes the control arm and k=1 the experimental treatment arm).

In randomized trials settings, the potential benefits of the experimental treatment over the control arm can be measured on the difference in outcome probabilities. Thus, one may consider that posterior probability that the experimental treatment is beneficial over the control defined at the ith trial is a measure of treatment effectiveness based on all the information recorded so far, that is given by $P\left( \pi_{k}-\pi_{0}>\Delta| y_{ki},n_{ki}, i=1,\ldots K \right)$, where $\Delta$defines some threshold of clinical interest. Based on the particular setting, values of $\Delta$ =0.05 and 0.10 were considered.

This posterior distribution $\left( \pi_{k}-\pi_{0}>\Delta| y_{ki},n_{ki}, i=1,\ldots K \right)$ is a difference of two beta distributions (of $\pi_{k}$ and $\pi_{0}$, respectively), which is no longer a beta distribution. This difference has been computed in relation to Appell’s hypergeometric functions; otherwise, a normal approximation has been proposed; however, when the difference between the sample proportions is small as possibly in our setting, the approximate probability is not equal to the exact probability (Kawasaki 2012). Thus, numerical integration were thus performed, as previously proposed (Cook 2005).

Secondly, we computed predictive probability as a measure of interest of conducting further trials. We first computed over time, the posterior predictive probabilities that given the results of all previous published trials, the next scheduled trial would achieve a risk difference of observed failures of at least 5% or 10% in favor of the experimental treatment. We also computed the required sample size of a new trial to reach a 95% coverage probability on average for the posterior credible interval (CrI) of 5% length for the risk difference of failure probabilities.

Estimates of the posterior mean and the endpoints of the 95% credible intervals were based on exact computations when available, or from the mean, 0.025, and 0.975 empirical quantiles of Markov Chain Monte Carlo (MCMC) samples. After an initial burn-in of 20,000, we operated another 30,000 iterations. The model was also run using 3 different chains of starting points to assess the influence that these points had on the model results. MCMC was performed with JAGS (Just Another Gibbs Sampler, <https://sourceforge.net/projects/mcmc-jags>) and the “R2jags” package (Su 2012); the “SampleSizeProportions" R package was used to the sample size computation.

**References**

- Cook JD. Exact calculation of beta inequalities. Technical Report 2005, UT MD Anderson Cancer Center Department of Biostatistics, UTMDABTR-005-05
- Kawasaki Y, Miyaoka E. A Bayesian inference of P(π1 > π2) for two proportions. Journal of Biopharmaceutical Statistics 2012;22: 425-437
- Su YS, Yajima M (2012) Package ‘R2jags’ v.0.03-8. http://cran.r-project.org/web/packages/R2jags/R2jags.pdf. Accessed 12 November 2012
